# Inhibition of Rac and ROCK Signalling Influence Osteoblast Adhesion, Differentiation and Mineralization on Titanium Topographies

**DOI:** 10.1371/journal.pone.0058898

**Published:** 2013-03-07

**Authors:** Paul D. H. Prowse, Christopher G. Elliott, Jeff Hutter, Douglas W. Hamilton

**Affiliations:** 1 Department of Anatomy, Graduate Program of Biomedical Engineering, The University of Western Ontario, London, Ontario, Canada; 2 Department of Cell Biology, Graduate Program of Biomedical Engineering, The University of Western Ontario, London, Ontario, Canada; 3 Department of Physics and Astronomy, The University of Western Ontario, London, Ontario, Canada; 4 Division of Oral Biology, Schulich School of Medicine and Dentistry, The University of Western Ontario, London, Ontario, Canada; INSERM U1059/LBTO, Université Jean Monnet, France

## Abstract

Reducing the time required for initial integration of bone-contacting implants with host tissues would be of great clinical significance. Changes in osteoblast adhesion formation and reorganization of the F-actin cytoskeleton in response to altered topography are known to be upstream of osteoblast differentiation, and these processes are regulated by the Rho GTPases. Rac and RhoA (through Rho Kinase (ROCK)). Using pharmacological inhibitors, we tested how inhibition of Rac and ROCK influenced osteoblast adhesion, differentiation and mineralization on PT (Pre-treated) and SLA (sandblasted large grit, acid etched) topographies. Inhibition of ROCK, but not Rac, significantly reduced adhesion number and size on PT, with adhesion size consistent with focal complexes. After 1 day, ROCK, but not Rac inhibition increased osteocalcin mRNA levels on SLA and PT, with levels further increasing at 7 days post seeding. ROCK inhibition also significantly increased bone sialoprotein expression at 7 days, but not BMP-2 levels. Rac inhibition significantly reduced BMP-2 mRNA levels. ROCK inhibition increased nuclear translocation of Runx2 independent of surface roughness. Mineralization of osteoblast cultures was greater on SLA than on PT, but was increased by ROCK inhibition and attenuated by Rac inhibition on both topographies. In conclusion, inhibition of ROCK signalling significantly increases osteoblast differentiation and biomineralization in a topographic dependent manner, and its pharmacological inhibition could represent a new therapeutic to speed bone formation around implanted metals and in regenerative medicine applications.

## Introduction

Titanium and its alloys have been used for bone-contacting implants for several decades due to their high biocompatibility, favourable mechanical properties and low corrosion [1], [2]. *In vivo*, titanium forms a direct interface with bone tissue through the process of osseointegration. By modifying the surface topography of the implant surface, formation of bone on titanium surfaces can be further enhanced. It is evident that cellular recognition of surface topography is extremely complex, and remains as of yet, poorly understood. Although many dental implant systems show great clinical success temporally [3], the time required for initial osseointegration and stability still remains a significant problem [4], particularly with the increase in demand for immediate loading [5].

Many different topographies are found commercially on dental implants, but moderately roughened topographies, produced through additive techniques such as titanium plasma spraying or subtractive techniques such as grit-blasting, acid etching or anodization are most common [2], [6]. Combinations of these treatments can also be used, as in the case of the **S**and-blasted, **L**arge grit, and **A**cid etched (SLA) topography produced by grit blasting followed by acid etching (Junker et al., 2009; Zhao et al., 2005). This technique results in a surface with hierarchical levels of roughness; large pores 20–50 μm occur as a result of sand blasting, and smaller micropits 0.5–2 μm in diameter result from HCl/H_2_SO_4_ treatment [7], [8]. The SLA topography has been shown to be osteoconductive *in vitro* [8]–[12], *in vivo* [13], [14], as well as in the clinical setting, with implant viability of 96.7% [15] and 99% [16] reported at 5 years.

*In vitro*, SLA increases osteoblast attachment, differentiation, and biomineralization, in comparison with smoother topographies, which promote adhesion formation, spreading, F-actin stressfibre formation and proliferation [8], [9]. SLA surfaces reduce F-actin stressfibre formation in osteoblasts [8], [12] at timepoints up to 24 hrs, unless the wettability of the surface and subsequent protein adsorption is increased [17]. Many studies have now demonstrated that osteoblast adhesion dynamics are an important regulator of signalling upstream of osteoblast commitment and differentiation [11], [17]–[26]. Therefore strategies, topographic or pharmacological, that directly target adhesion dynamics could potentially influence the rate of bone formation at earlier times after placement, potentially avoiding loosening.

Adhesion formation and F-actin reorganization, whether influenced by topography or not, are regulated by the Rho-family of GTPases [27], [28]. Focal contact formation and polymerization of actin at the cell periphery is regulated by Rac, whereas activation of RhoA and the downstream Rho-associated kinase (ROCK) is linked to F-actin stressfibres and development of focal contacts into focal adhesions [29]. RhoA mediation of stressfibre formation is associated with the localization of talin and vinculin to focal adhesions [30] which arise from Rac1-mediated focal contacts, illustrating the cross-talk and complexity of GTPase signalling [31].

Recent studies have shown that actin gene expression is downregulated in mineralizing, but not non-mineralizing osteoblasts [32]. Moreover, disruption of F-actin stressfibre formation using cytochalasin-D, although transient, results in increased alkaline phosphatase (ALP) and osteocalcin (OCN) secretion in MC3T3-E1 murine osteoblasts [33]. Based on their regulation of F-actin organization, we hypothesized that GTPase activity may play a role in the regulation of osteoblast differentiation. The functional significance of GTPases and F-actin reorganization in osteoblast differentiation on titanium with different topographical features has never been directly tested. In this study, we utilized two titanium topographies with different levels of roughness; pre-treated (PT), which is lightly acid etched to clean it, and SLA, which is blasted and etched producing much larger roughness. Specifically, we investigated how pharmacological inhibition of Rac and ROCK signaling influenced the adhesion, F-actin organization, migration, Runx2 nuclear translocation, differentiation and mineralization of rat calvarial osteoblasts (RCOs).

## Materials and Methods

### Ethics statement

All studies involving rats were performed in compliance with the University Council on Animal Care using protocols approved by the Animal Use Subcommittee at the University of Western Ontario specifically for this work.

#### Preparation and Characterization of titanium surfaces

SLA and PT Ti discs were prepared as previously described in Miron et al [34] and the surfaces have been extensively characterized by both Wieland et al [35] and Rupp et al [36]. Briefly, discs of 15 mm in diameter were punched out of a 1 mm thick commercially pure titanium grade 2 sheets. PT discs were degreased in acetone, followed by pickling with a mixture of dilute nitric and hydrofluoric acid and finally rinsed with reverse osmosis purified water. To create SLA surfaces PT surfaces were sand blasted with large grits (250–500 μm corundum particles), acid etched in a boiling mixture of HCl and H_2_SO_4_, and cleaned in dilute nitric acid followed by rinsing with pure water. The three-dimensional surface topography was quantitatively measured using confocal microscopy (μSurf, NanoFocus AG, Oberhausen, Germany). Images over an area of 798 μm ×798 μm were obtained with a 20× objective. Three-dimensional roughness parameters S_a_ (arithmetic mean deviation of the surface), S_t_ (maximum peak-to-valley height of the surface) and S_sk_ (skewness of the surface) were calculated using a moving average Gaussian filter with a cut-off wavelength of 30 μm. Three measurements each were made on three samples per group. The surface topography was qualitatively examined using scanning electron microscopy (Phillips XL30), using a tungsten filament as electron emitter and a secondary electron detector. Images were acquired with an acceleration voltage of 20 kV. The surface topography was qualitatively examined using scanning electron microscopy (Phillips XL30), using a tungsten filament as electron emitter and a secondary electron detector. Images were acquired with an acceleration voltage of 20 kV. To quantify levels of nanoroughness on PT surfaces, Atomic force microscopy was performed using a Digital Instruments Multimode AFM with NanoScope IIIa controller (Veeco, Santa Barbara, CA). Measurements were either made in height mode, with a scan direction of 0° (along cantilever axis), while recording the trace and retrace height information (measured in nm), or in lateral-force mode, with a scan direction of 90°, while recording the trace and retrace lateral force data (the A-C signal, measured in V).

#### Osteogenic Cell Culture

Rat Calvarial Osteoblasts (RCOs) were isolated from 0–5 days old Sprague Dawley® rats by enzymatic digestion as previously described [23], [24], [34], [37]. Briefly, parietal and occipital bones were dissected, minced and 700 units/ml of type I collagenase (C0130, Sigma-Aldrich, St. Louis, MO, USA) was used to release the RCOs from the bone, with the first supernatant discarded. Cells were plated in tissue culture flasks using α-MEM supplemented with antibiotics and antimycotics (100 μg/ml penicillin G, 50 μg/ml gentamicin, 25 μg/ml amphotericin B, and 10% fetal bovine serum (Gibco, Grand Island, NY, USA)). Cultures were maintained in a 5% CO_2_ atmosphere with 100% relative humidity at 37°C.

For experiments, cells were released from 75 cm^2^ tissue culture plastic flasks using a 0.05% trypsin and 0.2 g/L EDTA·4Na solution and counted using a Beckman Coulter^TM^ Multisizer^TM^ 3 Coulter Counter® (Mississauga, Ontario, Canada). RCOs were seeded at 3×10^4^ cells/cm^2^ for real-time RT-qPCR analysis and at 1×10^4^ cells/cm^2^ for immunofluorescence. For experiments lasting longer than 24 hours, cells were supplemented at seeding with 50 µg/ml of ascorbic acid and 2 mM β-glycerol phosphate to induce differentiation. Rac activity was inhibited using NSC23766 (Tocris Biosciences, Ellisville, MO, USA) reconstituted in sterile water and used at a final concentration of 100 mM. ROCK signaling was inhibited using Y-27632 (Cayman Chemical, Ann Arbor, MI, USA). Y-27632 was reconstituted in dimethyl sulfoxide (DMSO) and used at a final concentration of 10 μM. The NSC23766 and Y-27632 inhibitors were added at the time of cell seeding and media was changed daily to ensure continuous presence and activity of the inhibitors. To assess bone nodule formation, tetracycline (Sigma-Aldrich) was added to the media at 5 μg/ml. Images were imported into imageJ, and the intensity of staining and the numbers of nodules per surface counted calculated.

#### Assessment of Rac1 and ROCK activity

To assess the efficacy of NCS23766 and Y27632 on inhibition of Rac1 and ROCK respectively, osteoblasts were cultured on tissue culture plastic to ensure sufficient protein could be extracted for the assays. Cells were grown in the presence of the inhibitors for 1 and 3 weeks, with osteogenic media serving as a control. Rac1 activity was assayed using G-LISA™ Rac1 assay biochem kit™ (Cytoskeleton Inc, Denver, CO) according to manufacturers instructions. ROCK activity was quantified using the Rho-associated kinase activity assay (Millipore). Activity was normalized to GAPDH to ensure equal protein loading.

#### Fluorescent Microscopy

Cells designated for immunofluorecent labeling were fixed in 4% paraformaldehyde in phosphate buffered saline (PBS) and permeabilized with 0.1% Triton-X 100 in PBS for five minutes. Non-specific antibody interactions were blocked using 3% bovine serum albumin (BSA) in PBS for 30 minutes prior to the addition of the primary antibody. Samples were incubated with primary antibodies to vinculin (MAB3574, Millipore, Temecula, CA, USA), osteocalcin (AB10911, Millipore), and bone sialoprotein (Gift from Dr Harvey Goldberg) Runx2 (sc-10758, Santa Cruz Biotechnology, Santa Cruz, CA) at a 1∶100 dilution. All antibodies were diluted in 0.5% BSA in PBS. After a 1 hour incubation with the primary antibody, the surfaces were rinsed three times and then incubated with a 1∶200 dilution of Alexa Fluor® 488 goat anti-mouse IgG (A11001, Invitrogen Canada Inc., Burlington, ON, CAN) for visualization of the primary antibody and a 1∶100 dilution of Rhodamine phalloidin (R415, Cytoskeleton Inc.) for visualization of filamentous actin. Nuclei were counterstained using 4’,6 diamidino-2-phenylindole (DAPI) using Vectashield Mounting Medium (Vector Labs, Burlington, Ontario, Canada). To visualize bone nodule formation by tetracycline incorporation, an illumination wavelength of 488 nm was used as previously described [8]. Images were captured from each surface on an AxioScope fluorescence microscope (Carl Zeiss Canada, Toronto, ON, CAN) using an Axiocam digital camera and AxioImager software. To assess focal adhesions, images were exported in the Tagged Image File Format (TIFF). Images were thresholded to quantify the size and number of focal adhesions using ImageJ (Bethesda, MD, USA). For quantification of Runx2 nuclear translocation, separate images were captured of antibody labeling and nuclear area. To be included in the analysis, Runx2 staining had to conform within the boundaries of the nucleus. Positive nuclei were counted using imageJ and data imported into Graphpad Prism version 5.00 (La Jolla, CA) for analysis. For BSP and OCN staining, images were imported into ImageJ. The level of threshold was set using the first image and then applied to all images, with data exported into Graphpad Prism for analysis.

#### Real-time RT-qPCR analysis

Total RNA was extracted using TRIZOL® reagent at 1 and 7 days according to the manufacturer’s recommendations. Probes for osteocalcin (*Bglap*, Rn01455285_g1), bone sialoprotein (*iBsp*, Rn01450118_ml), bone morphogenetic protein-2 (*Bmp2*, Rn 00567818_ml) Runx2 (*Runx2*, Rn01512296_ml) and ATF-4 (*Atf4*, Rn00824644_g1) were obtained from Applied Biosystems^TM^ (Carlsbad, CA, USA). RT-PCR was run on the extracted RNA using a final reaction volume of 15 μl per well. TaqMan® One-Step RT-PCR Master Mix Reagents was added to 50 ng of RNA per well in order to obtain the final reaction volume. Each well contained RNA from one titanium surface. All experiments were completed in quadruplicate and repeated three independent times. Gene expression levels were reported relative to *18S* levels and normalized to day 1 PT control cells.

#### Statistical Analysis

All experiments were performed with cells from three independent isolations, and with at least 3 replicates within each independent experiment. For Rac1 and ROCK activity assays, treatments were analyzed via one-way ANOVA with a Bonferroni post-test was used to test for significance. For all other experiments, two-way ANOVA was used as the statistical test with a Bonferroni post-hoc test. All statistical analysis was performed using Graphpad Prism version 5.00 (La Jolla, CA). p<0.05 was considered significant.

## Results

### Surface Characterization

**Figure 1:**
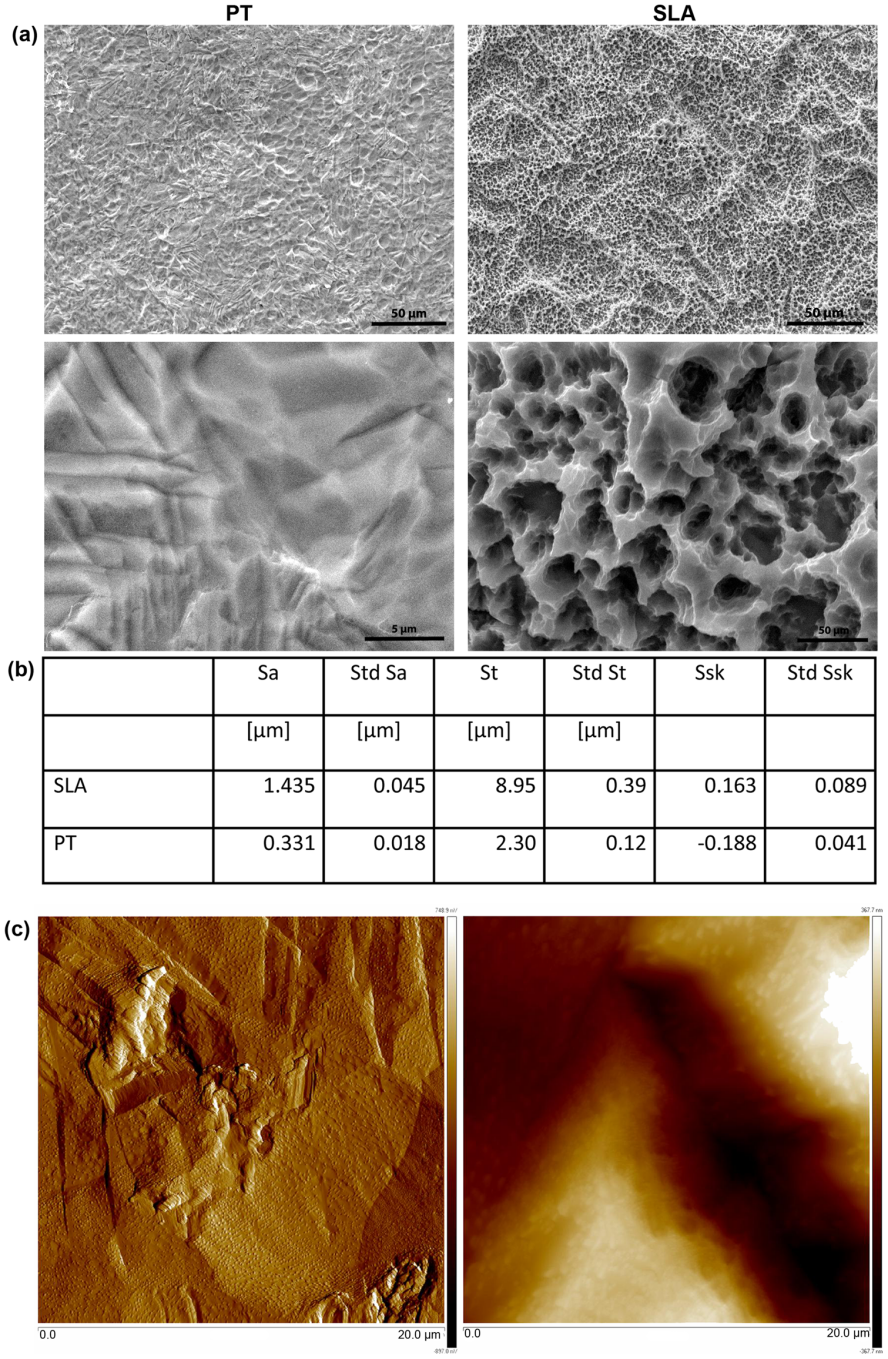
Characterization of the surfaces used in the study. (a) SEM micrographs of pre-treated (PT) and SLA surfaces. In (b) surface roughness parameters of the three-dimensional surface topography of PT and SLA quantitatively measured using confocal microscopy. For detailed description, see materials and methods. (c) AFM micrographs of the PT surface.

SEM micrographs of the surfaces are shown in figure 1a. The S_a_ of the SLA topography was 1.435 µm in comparison with the PT, which was 0.331 µm (Figure 1b). The average peak to peak height (S_t_) (defined as the height difference between the highest and lowest points) for PT was 2.3 µm and for SLA was 8.95 µm. As expected from the SEM micrographs, the S_sk_ of PT was negative indicating a predominance of smooth valleys, while SLA had a positive skew, which is characteristic of surfaces with peaks. AFM analysis of PT surfaces revealed that the surfaces have significant nanoroughness (Figure 1c). The surfaces had a mean roughness depth of 715.20 nm and a mean square average of the roughness profile (RMS) of 136±35 nm.

### Temporal Analysis of Rac1 and ROCK inhibition of Osteoblasts

**Figure 2:**
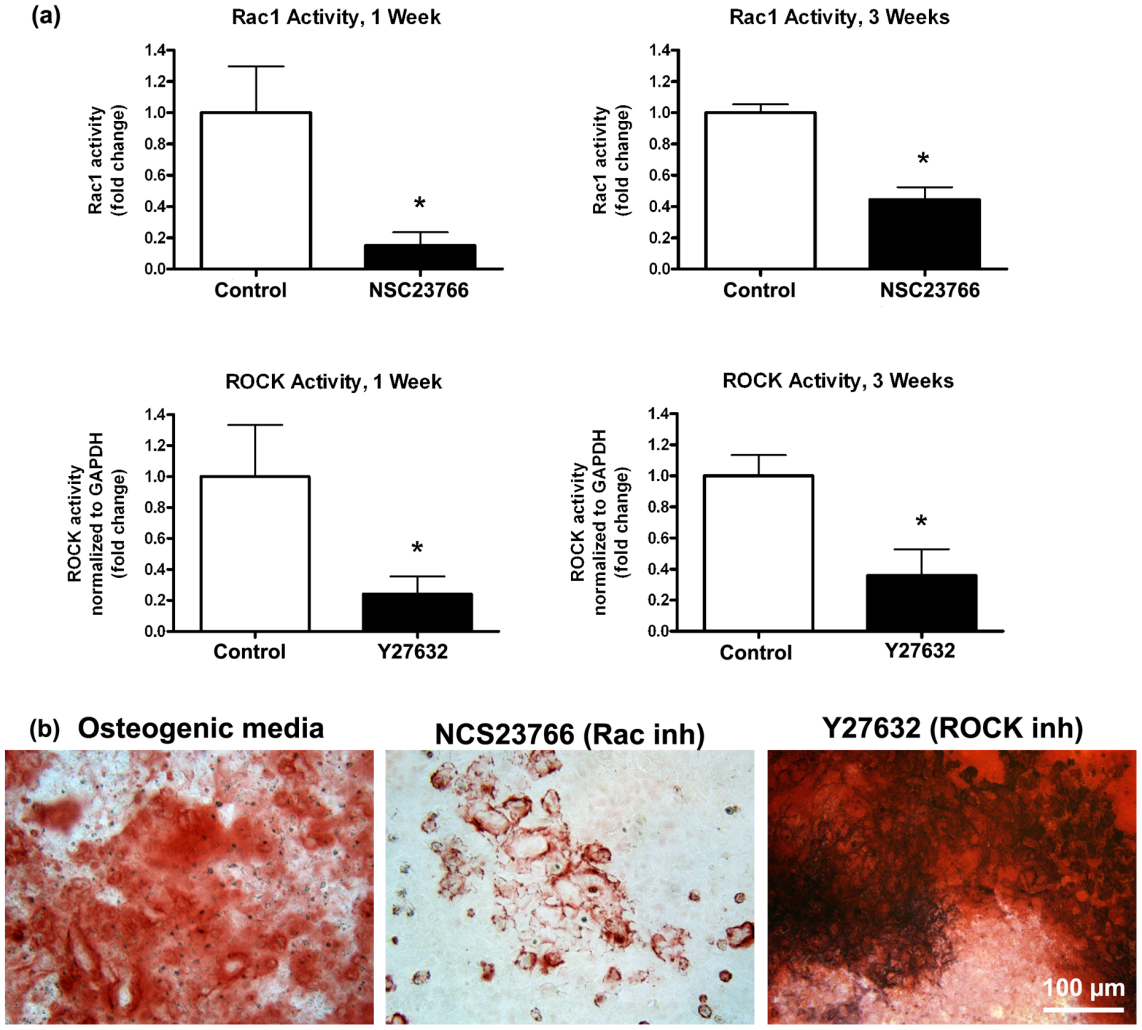
Influence of NCS23766 and Y27632 on the activity of Rac1 and ROCK on tissue culture plastic. Studies were run for 1 week and 3 days to quantify the temporal suppression of the inhibitors. NCS23766 significantly reduced Rac1 activity and Y27632 inhibited the activity of ROCK. Alizarin red staining of wells demonstrated increased mineralization in the presence of ROCK inhibitors. Treatments were analyzed via one-way ANOVA with a Bonferroni post-test (* denotes significance of p<0.05 between treatments).

At 1 and 3 weeks, NCS23766 significantly suppressed activity of Rac1 compared to osteogenic media alone (Figure 2). Similarly, Y27632 significantly inhibited ROCK activity. Staining of wells revealed a decrease in alizarin red staining in the presence of NCS23766, but an increase in the presence of Y27632 compared to osteogenic media alone.

### Effect of Rac and ROCK inhibition on Focal Adhesion formation

**Figure 3:**
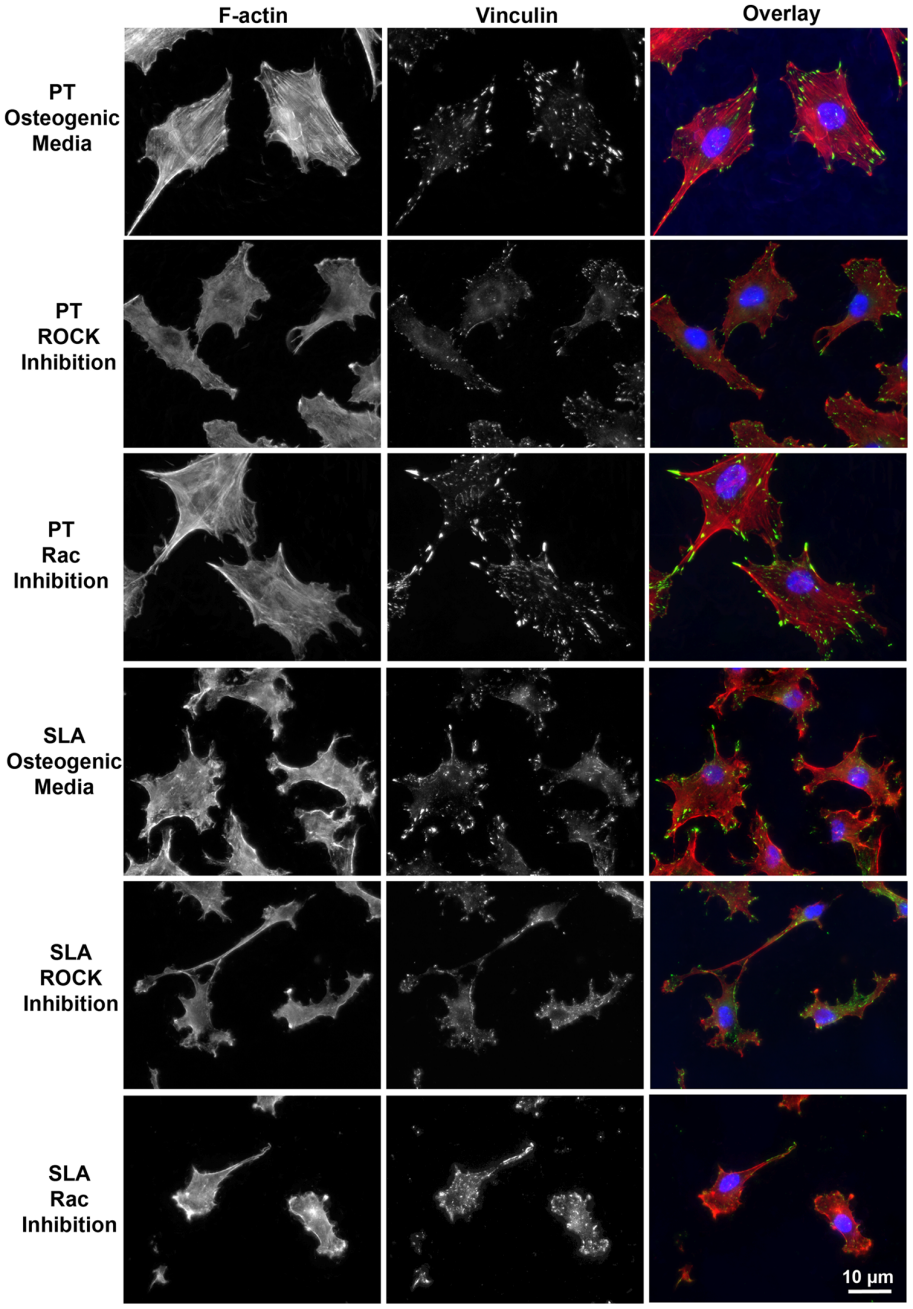
Immunofluorescent detection of adhesions and F-actin organization in osteoblasts cultured on PT and SLA surfaces with and without Rac and ROCK inhibition at 24 h. Osteoblasts form stressfibres on PT, but not SLA. ROCK inhibition disrupts stressfibre formation in osteoblasts on both PT surfaces. Cells were stained for vinculin (green), F-actin (red), and nuclei (blue).

**Figure 4:**
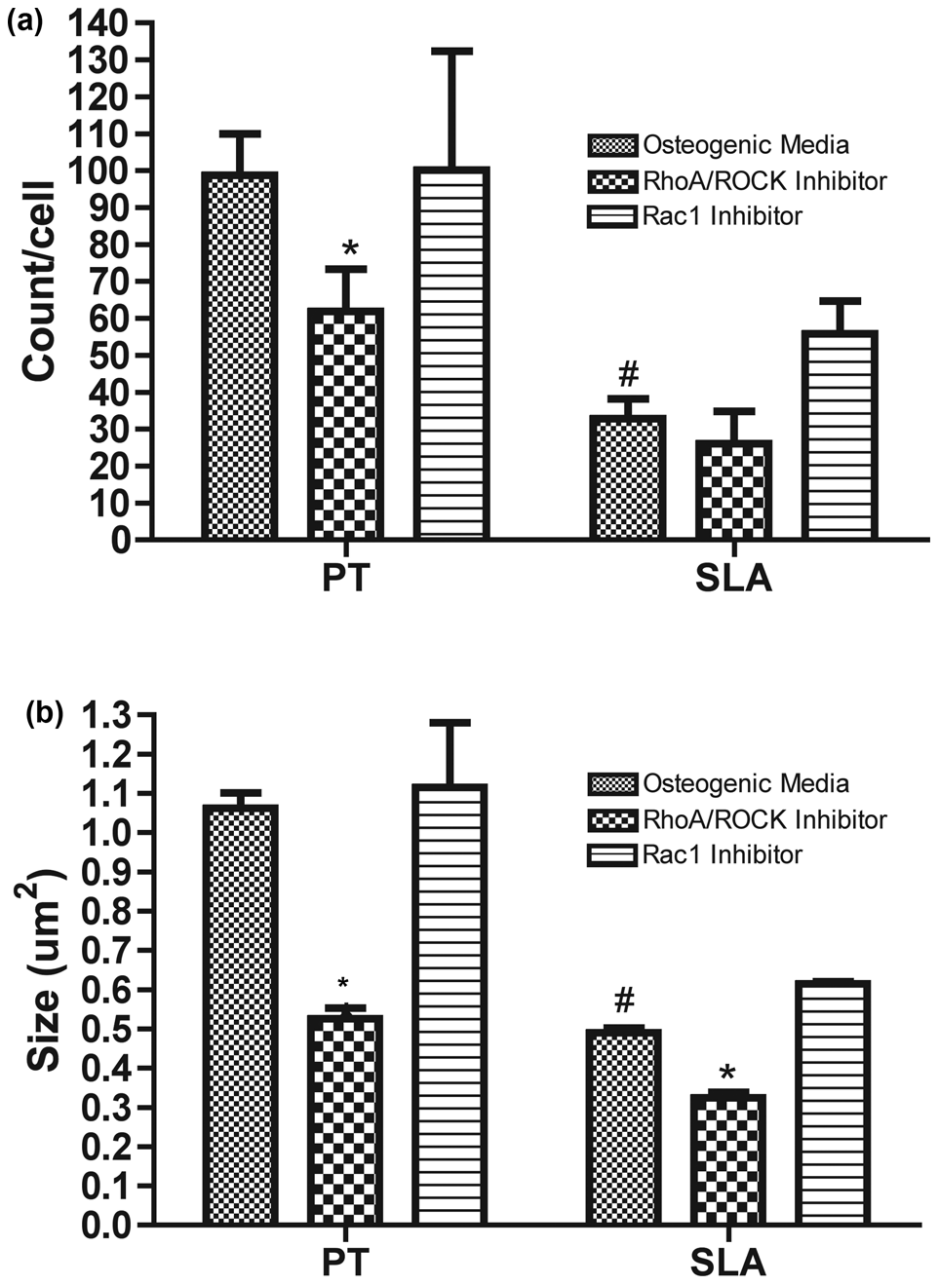
Influence of topography, Rac and ROCK inhibition on (a) adhesion number and (b) adhesion size. Graphs represent mean ± SEM of 3 independent experiments completed in triplicate. Treatments were analyzed via two-way ANOVA with a Bonferroni post-test (# denotes significance of p<0.05 between PT and SLA, * denotes significance of p<0.05 between treatments within a surface type).

FA formation and F-actin organization were influenced by both surface topography, as well as the addition of Rac and ROCK inhibitors at 24 hrs post-seeding (Figures 3, 4). Under all experimental conditions, adhesion size was consistent with the formation of focal complexes as opposed to mature FAs. RCOs cultured in osteogenic media on SLA showed a significant reduction in adhesion number and size compared with control cells on PT (p<0.05) (Figure 4A, B). On PT surfaces, ROCK, but not Rac, inhibition significantly reduced the average number of adhesions and their size in each cell (p<0.05) (Figure 4A, B). ROCK inhibition significantly reduced adhesion size in RCOs on both PT and SLA surfaces (p<0.05) (Figure 3, 4B).

### Effect of Rac and ROCK on Osteogenic mRNA expression

**Figure 5:**
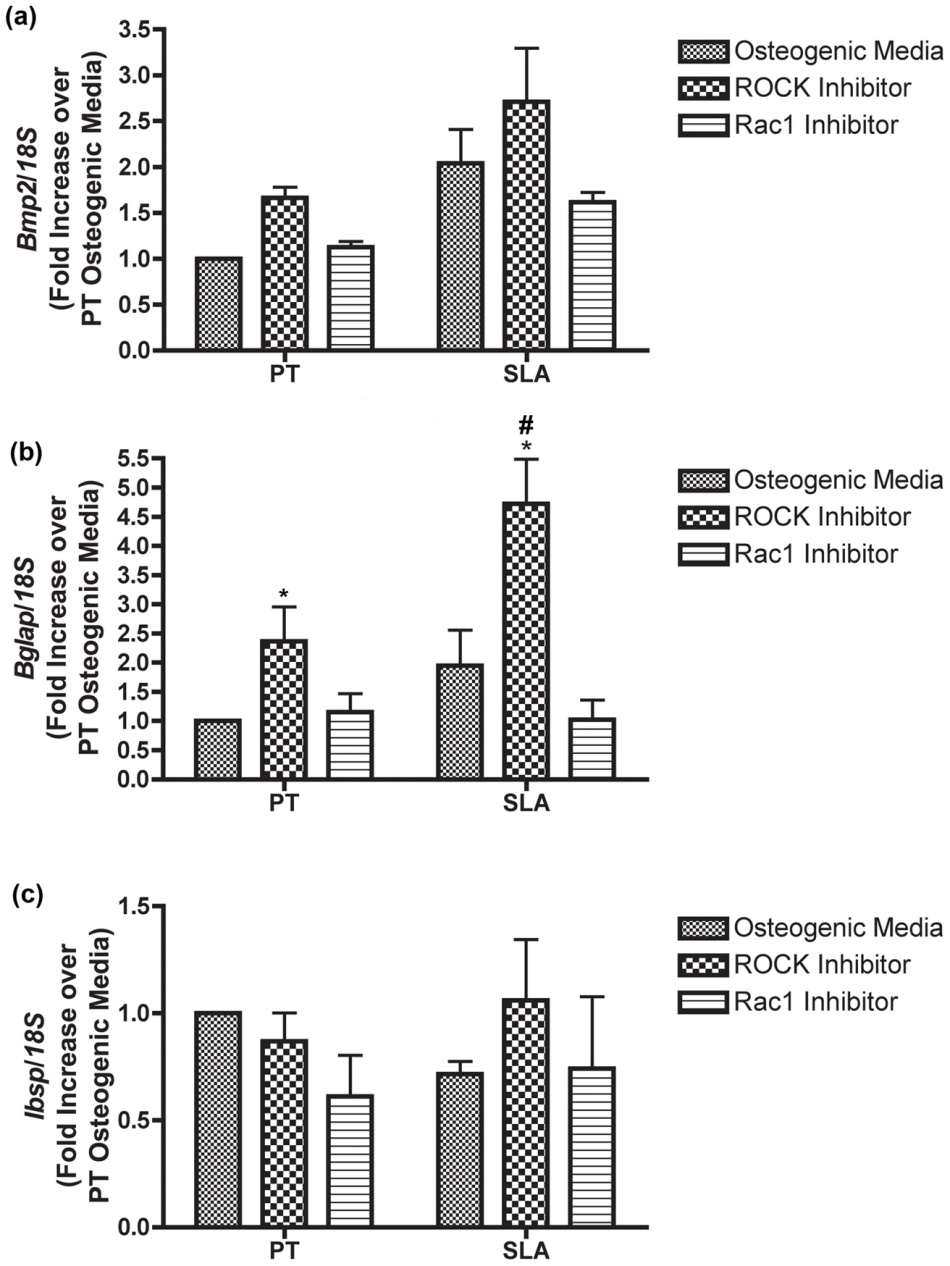
Influence of topography, Rac1 and ROCK inhibition on expression of (a) BMP-2, (b) osteocalcin and (c) bone sialoprotein in osteoblasts at 24 h post seeding. ALP mRNA levels were significantly increased on PT by both inhibitors, but only ROCK had an effect on SLA. No significant differences were observed in BMP-2 or BSP levels under any treatment, but ROCK inhibition significantly increased osteocalcin levels on both PT and SLA. Graphs represent mean ± SEM of 3 independent experiments completed with 4 replicates per experiment. Treatments were analyzed via two-way ANOVA with a Bonferroni post-test (# denotes significance of p<0.05 between PT and SLA, * denotes significance of p<0.05 between treatments within a surface type).

**Figure 6:**
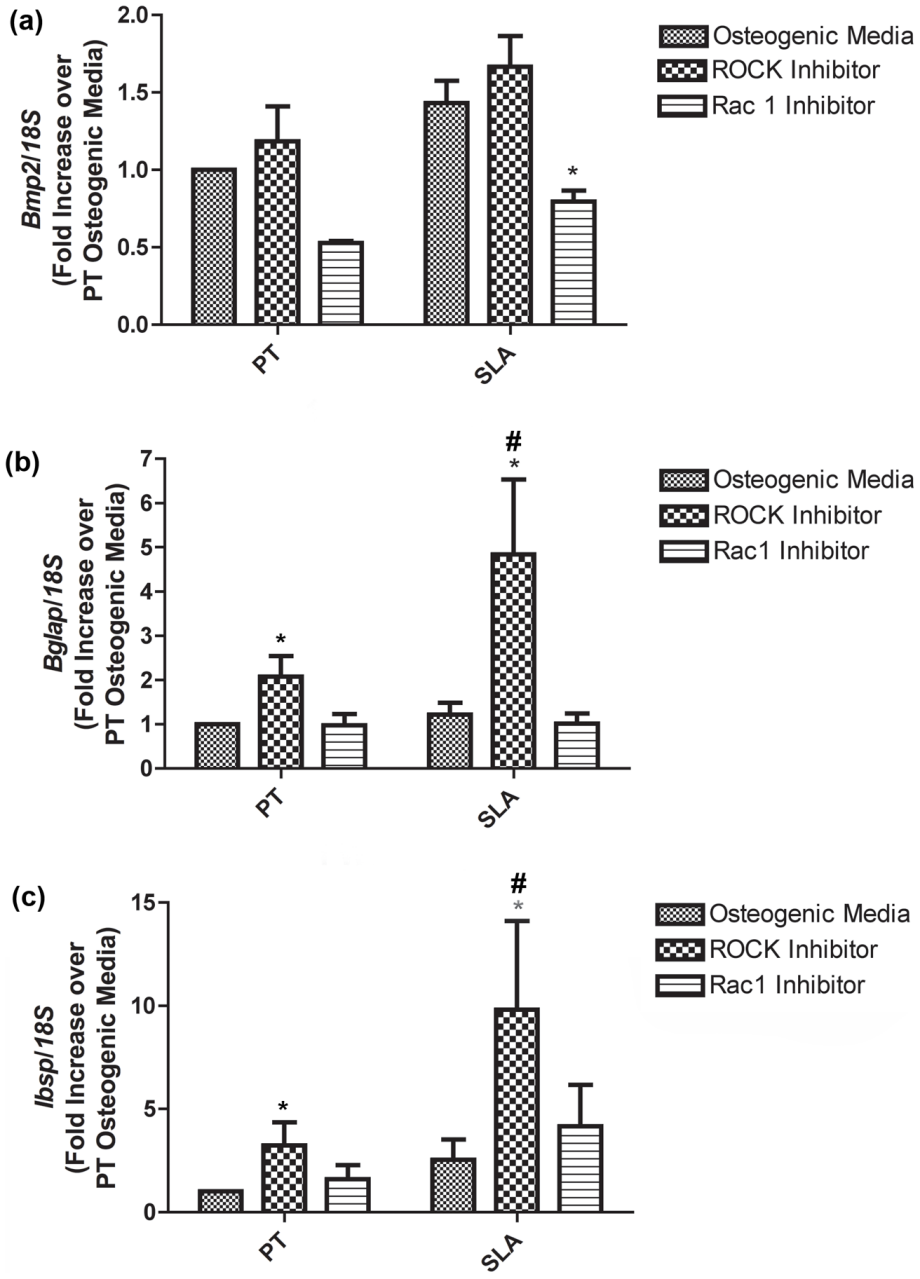
Influence of topography, Rac1 and ROCK inhibition on expression of (a) BMP-2, (b) osteocalcin and (c) bone sialoprotein in osteoblasts at 7 days post seeding. ROCK inhibition significantly increased osteocalcin and BSP mRNA levels on both PT and SLA compared to control surfaces. Graphs represent mean ± SEM of 3 independent experiments completed with 4 replicates per experiment. Treatments were analyzed via two-way ANOVA with a Bonferroni post-test (# denotes significance of p<0.05 between PT and SLA, * denotes significance of p<0.05 between treatments within a surface type).

**Figure 7:**
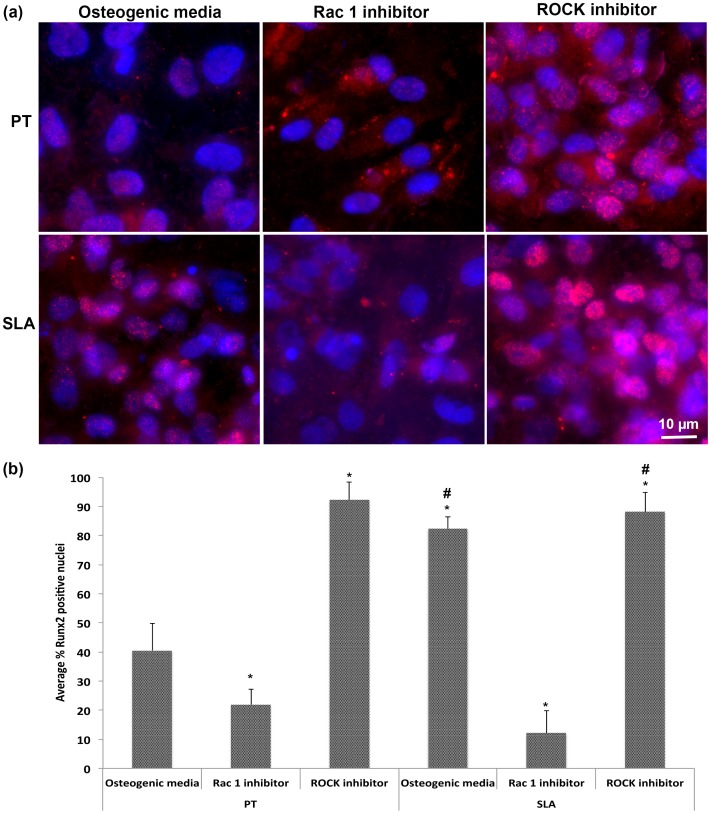
Rac1 and ROCK inhibition influence localization of the osteogenic transcription factor, Runx2. (a) At 1 week, cells were labeled with specific antibodies to Runx2, and (b) quantification of nuclear translocation. Rac1-inhibition reduced nuclear localization of Runx2 on both PT and SLA compared to controls although it was still found in the cytoplasm. ROCK inhibition increased nuclear localization of Runx2 on both PT and SLA compared to controls and it was also found in the cytoplasm. Treatments were analyzed via two-way ANOVA with a Bonferroni post-test (# denotes significance of p<0.05 between PT and SLA, * denotes significance of p<0.05 between treatments within a surface type).

The relative gene expression of BMP-2, osteocalcin, BSP, ATF-4 and Runx2 in RCOs was assessed at 1 and 7 days post seeding (Figure 5, 6). BMP-2 mRNA levels did not significantly change in any condition at day 1 (Figure 5A). Osteocalcin was significantly upregulated in RCOs cultured on both PT and SLA in the presence of ROCK inhibitors at day 1 (p<0.05) (Figure 5B). No significant difference in BSP mRNA levels was observed under any condition (Figure 5C). Inhibition of Rac activity significantly decreased BMP-2 mRNA levels on SLA, but not PT at 7 days (Figure 6A) (p<0.05). Inhibition of ROCK resulted in significant increases in osteocalcin expression on both PT and SLA at 7 days (p<0.05) (Figure 7B), with levels significantly higher on SLA than PT. ROCK inhibition significantly increased BSP mRNA levels on SLA (p<0.05), but not on PT (Figure 6C). The transcription factors ATF-4 and Runx2 showed no change in mRNA levels under any of the tested conditions compared with controls (data not shown).

### Effect of Rac and ROCK on Runx2 Nuclear Translocation

After 1 week of culture, Runx2 was translocated to cell nuclei in RCOs cultured on both PT and SLA, although it was also present in the cytoplasm (Figure 7). Inhibition of Rac activity reduced nuclear translocation of Runx2 on SLA and PT (p<0.05) (Figure 7A, B). In contrast, ROCK inhibition increased nuclear localization of Runx2 on both PT and SLA over that observed in control osteoblasts (p<0.05) (Figure 7A, B).

### Effect of Rac and ROCK on RCO Mineralization

**Figure 8:**
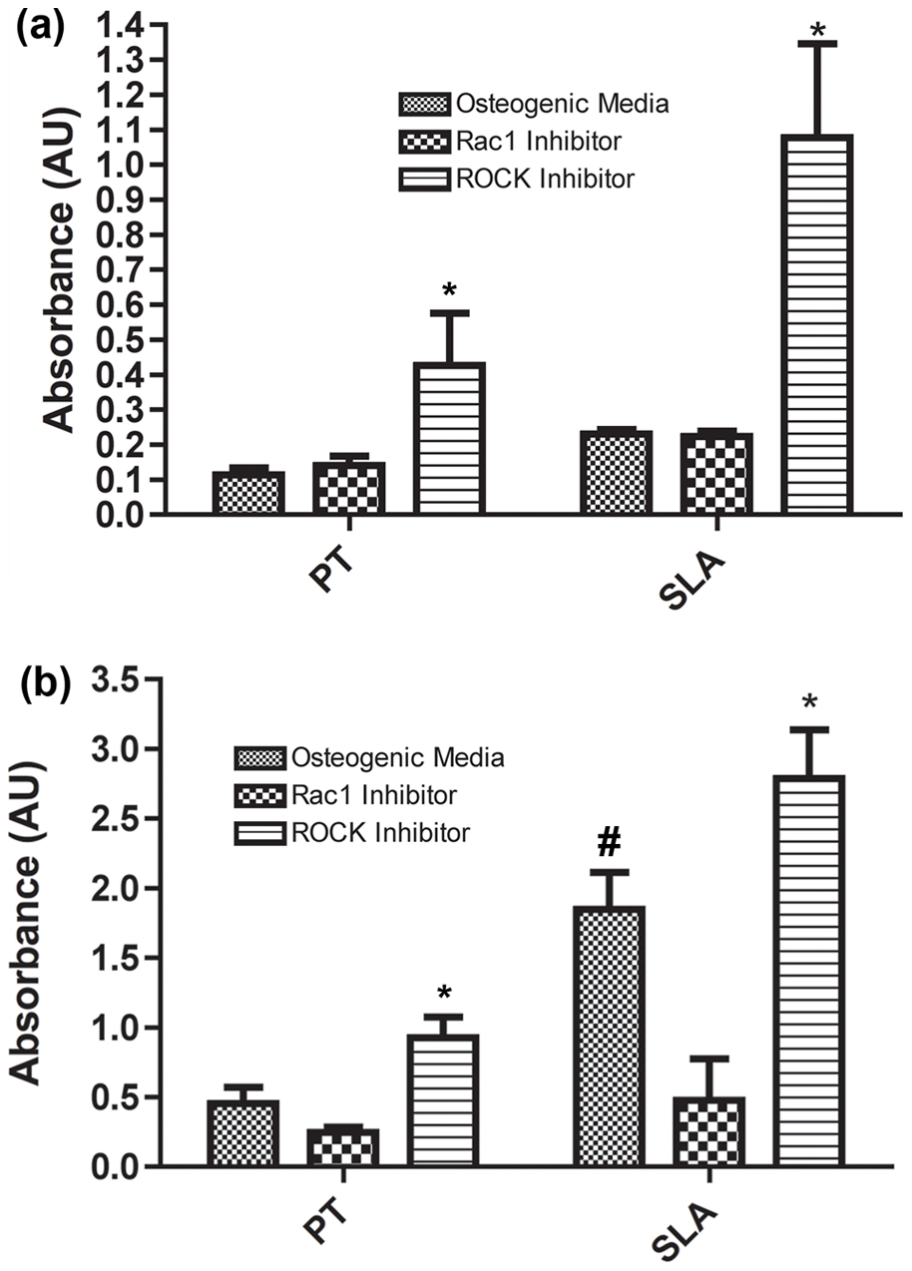
Topography, Rac1 and ROCK inhibition synergistically affect osteoblast biomineralization. (a) Quantification of the intensity of tetracycline labelling and (b) number of nodules demonstrated that ROCK inhibition significantly increased mineral deposition by osteoblasts on both PT and SLA. Images are representative of triplicates from three experiments. Treatments were analyzed via two-way ANOVA with a Bonferroni post-test (# denotes significance of p<0.05 between PT and SLA, *denotes significance of p<0.05 between treatments within a surface type).

**Figure 9:**
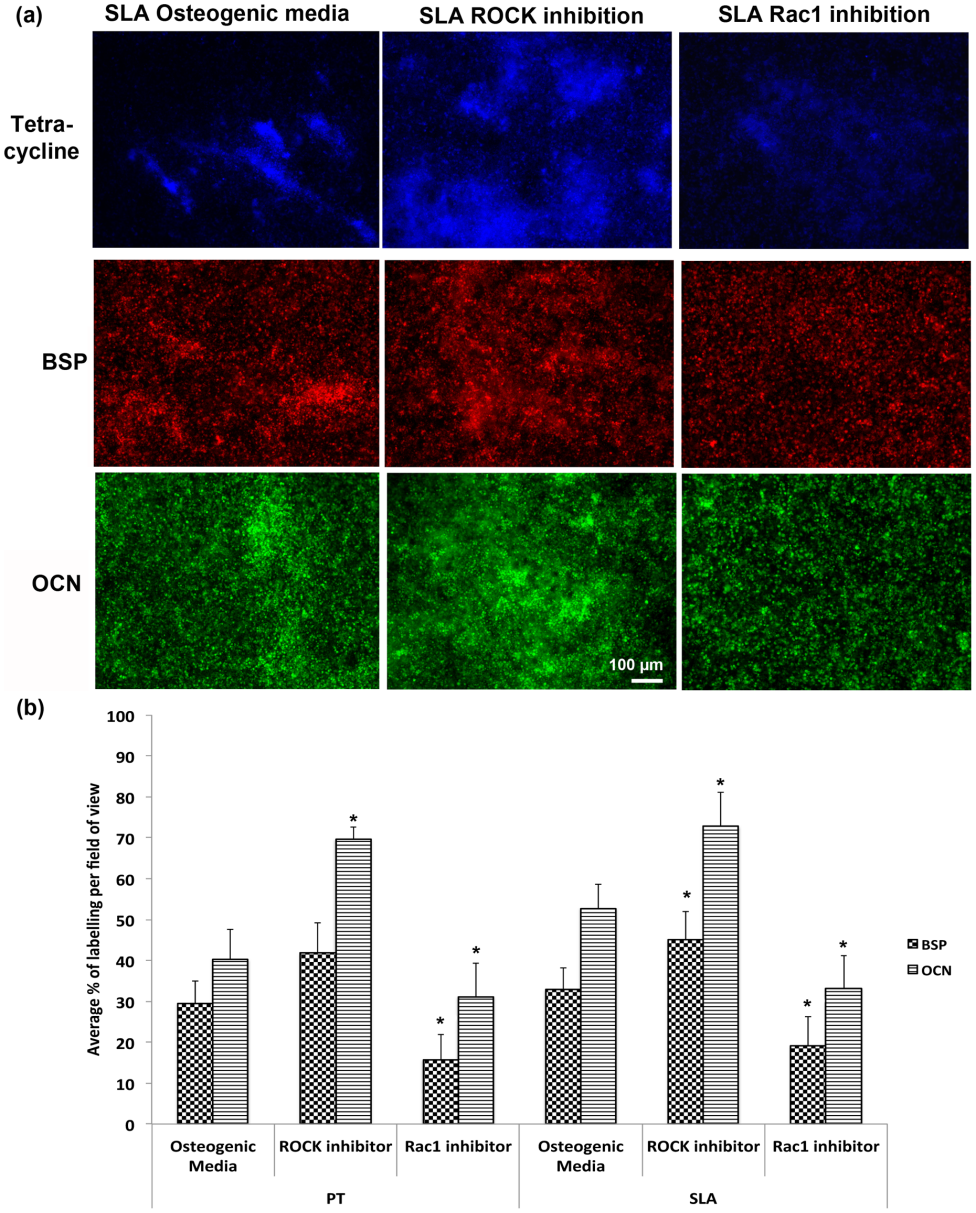
Topography, Rac1 and ROCK inhibition synergistically affect osteoblast deposition of BSP and OCN. (a) representative images of tetracycline, osteocalcin and BSP on SLA surfaces at 4 weeks post seeding (PT showed same pattern, not shown). (b) Quantification of BSP and OCN labeling at 4 weeks post seeding. Images are representative of triplicates from three experiments. Treatments were analyzed via two-way ANOVA with a Bonferroni post-test (# denotes significance of p<0.05 between PT and SLA, * denotes significance of p<0.05 between treatments within a surface type).

To assess whether the changes in mRNA levels and transcription factor localization resulted in changes in mineralization, we quantified mineral deposition using tetracycline incorporation at 4 weeks post seeding. Intensity of tetracycline staining was significantly higher on control SLA than on PT (Figure 8A) (p<0.05). ROCK inhibition significantly increased the staining intensity on both PT and SLA (p<0.05), but was higher on SLA. The number of bone nodules followed the same pattern, with ROCK inhibition significantly increasing nodule number on both PT and SLA (p<0.05) (Figure 8B). Specific antibody staining for osteocalcin and BSP demonstrated increased presence of both proteins on both PT and SLA with ROCK inhibition (Figure 9A, B). Quantification of labelling showed that ROCK inhibition significantly increased the area of staining for OCN and BSP on SLA, but only OCN on PT (p<0.05) (Figure 9B). Inhibition of Rac significantly reduced the area of labelling of BSP, but not OCN on PT (p<0.05) (Figure 9B). On SLA, Rac inhibition reduced the area of staining of both BSP and OCN compared to controls (p<0.05) (Figure 9B).

## Discussion

Topographical-induced adhesion and morphological changes influence osteoblast migration, differentiation, and mineralization on titanium [11], [18], [23], [38], but the molecular pathways underlying these observations are still poorly understood. Osteoblasts cultured on smooth surfaces such as PT form large numbers of adhesions and have well-developed F-actin stressfibres, but show slower and/or reduced differentiation. Osteoblasts cultured on rough topographies such as SLA show higher levels of differentiation, but they form fewer adhesions and the presence of stressfibre networks is disrupted (Figure 3 and [8]). These differences in F-actin organization and adhesions observed in osteoblasts on PT and SLA surfaces are indicative of differences in GTPase activation [27], [39]. Based on these observations, we hypothesized that Rac and ROCK pathways will play a role in the regulation of osteoblast adhesion, differentiation and biomineralization.

To investigate the roles of Rac and ROCK signaling in osteoblast response to PT and SLA topographies, we utilized the well-characterized pharmacological inhibitors NSC23766 (Rac) and Y27632 (RhoA-ROCK). NSC23766 is a cell-permeable inhibitor of Rac activation through inhibition of the Rac-specific guanine exchange factors TrioN and Tiam 1, both of which catalyze GDP/GTP exchange. With regards to specificity, NSC23766 likely also inhibits Rac2 and Rac3, but Rac2 is mainly expressed in hematopoietic lineages and Rac3 is associated with neuronal development. NSC23766 has no effect on cdc42 or RhoA activation. To inhibit ROCK indirectly, we used Y27632, which is a non-isoform-selective inhibitor that targets the ATP-dependent kinase domains of both ROCK1 and ROCK2. Essentially Y27632 works by competing with ATP for binding to the kinases domains of the ROCKs. Based on previous studies [31], [40]–[42], these inhibitors are specific to delineate the roles of Rac and ROCK in osteoblast response to alterations in substratum topography.

The most prominent finding from our study is that inhibition of ROCK signaling in osteoblasts significantly increases osteocalcin (PT and SLA) and bone sialoprotein (SLA) expression at 1 week, and biomineralization at 4 weeks. Recent evidence suggests that ROCK inhibition results in an increase in Wnt signalling in MC3T3 osteoblasts cultured on titanium [43]. Beta-catenin activation, a molecule involved in Wnt canonical signalling, is increased in MC3T3 cells cultured on SLA compared to PT, but ROCK inhibition significantly increased ALP and osteocalcin mRNA levels on PT at 3 days. Such dramatic effects were not evident in ROCK inhibited cells on SLA, which they attributed to differences in cytoskeletal tension induced by the different topographies. Interestingly they found no significant influence of ROCK inhibition on osteogenic gene expression of osteoblasts cultured on SLA [43]. In our study, osteocalcin was significantly increased at both 1 day and 1 week on both PT and SLA in the presence of ROCK inhibitors, with mRNA levels always significantly higher on SLA than PT. We suggest that ROCK inhibition likely increases the speed of osteoblast terminal differentiation by modulating cytoskeletal tension as Galli et al demonstrated, but other mechanisms are also likely involved other than Wnt signaling. There are several targets downstream of ROCK and these will require further examination in the context of osteoblast gene expression and topographical cues [44].

The measured changes in gene expression in osteoblasts induced by ROCK inhibitors were mirrored by increases in biomineralization at 4 weeks on both PT and SLA topographies, assessed through tetracycline incorporation. Inhibition of Rac activity reduced nuclear translocation while ROCK inhibition increased nuclear translocation of the osteocalcin transcription factors Runx2. Interestingly, Runx2 gene expression levels did not change under Rac or ROCK inhibition (data not shown), suggesting that upregulation of the gene is not required and it is nuclear accumulation of the protein that is altered. ROCK inhibition has been previously demonstrated to increase osteocalcin synthesis and biomineralization of mouse calvarial osteoblasts [45], [46] and the stromal cell lines C3H10T1/2 and ST2 [47] cultured on tissue culture polystyrene. Furthermore, Yoskihawa et al, 2009, observed that ROCK inhibition induced ectopic bone formation in response to rhBMP-2 [48]. Interestingly, ROCK inhibition significantly increases smooth muscle cell alkaline phosphatase (ALP) activity and calcification of rat aorta organ cultures [49], as well as mineralization of chondrogenic cells [50]. These studies suggest that ROCK 1 and ROCK 2 negatively regulate gene expression and mineralization in differentiated cell types, which is consistent with the findings from this study. This is in contrast to several studies that have shown that osteogenic differentiation of mesenchymal stem cells through modulation of nanotopography or matrix stiffness requires ROCK activation [50]–[54]. It therefore appears likely that ROCK signalling is required for MSC commitment and differentiation to osteoblasts, but terminal differentiation of osteoblasts requires suppression of ROCK. However, most of the studies on osteoblasts/ROCK have been murine, whereas MSC to osteoblasts studies have been done with human cells, so the possibility exists that signalling differences and requirement of Rac/ROCK could result from cell source.

Inhibition of ROCK signalling resulted in a significant reduction in FA formation and disruption of F-actin stressfibres, particularly in cells on PT. Rac1 and Cdc42 stimulate the formation of focal contacts, with ROCK regulating the subsequent formation of actin stressfibres bundles and the development of mature FAs. On SLA surfaces, osteoblasts form adhesions consistent in size with focal complexes, larger mature FAs being relatively rare [8], [34]. On PT, which contains significant nanoroughness, osteoblast differentiation is higher than on a planar smooth surface, and fewer mature adhesions are formed (>5 µm). The relative importance on topographic regulation of FA size and stability in osteoblasts has received much attention. We have previously shown that FA-mediated signaling is activated on microfabricated topographies that enhance osteoblast differentiation [23], [24]. Other studies have correlated adhesion size with osteoblast gene expression [19], but the results from this current study suggest that it is more likely to be which signalling cascades are activated, rather than the actual size or maturity of the FAs, that is the governing factor.

Although the current SLA topography has been a very effective topography for promoting osseointegration clinically [16], [55]–[57], our results demonstrate that considerable enhancements could be made to accelerate bone formation and primary fixation beyond the effects of the topography alone at early timepoints. We have previously shown that pre-adsorption of enamel matrix derivative to PT and SLA topographies enhanced osteoblast differentiation [34]. Moreover, the adsorption of the enamel proteins negated the influence of the topography, with bone sialoprotein and osteocalcin mRNA levels increased to similar levels in osteoblasts cultured on both EMD pre-coated PT and SLA. Therefore, although topographical cues are important determinants of implant integration, it is clear that protein deposition and soluble factors can also strongly influence the rate of bone formation.

The effectiveness of ROCK inhibition on accelerating osteoblast differentiation and mineralization has huge implications not only for enhancing implant integration, but also for bone tissue engineering applications. In 1986, Bellows et al. demonstrated the necessity of ascorbic acid (AA) and beta-glycerol phosphate (BGP) for matrix secretion and mineralization of osteoblasts respectively *in vitro* [37]. Since it was defined, osteogenic media has consisted exclusively of AA and BGP, although dexamethasone is often also included as it increases osteoblast differentiation *in vitro* [58]. Based on the results of this study, we propose that a ROCK inhibitor could be considered as a standard additive to osteogenic media to accelerate bone formation *in vitro*. One of the major issues facing patient-specific tissue regeneration is the amount of time required for the patient’s cells to migrate into, proliferate, differentiate, and produce matrix and mineral within a scaffold *ex vivo*. The use of a ROCK inhibitor could accelerate osteoblast infiltration, differentiation, and mineralization in both *ex vivo* (addition to media in bioreactors) and *in vivo* (systemic administration) seeded scaffolds for bone tissue regeneration. This would ultimately reduce the amount of time required to produce patient-specific tissues suitable for implantation. However, if mesenchymal stem cells were used as a cell source, it is likely that ROCK inhibitors may only function in temporal manner beyond initial scaffold colonization/cell proliferation and MSC commitment to the osteogenic lineage.

## Conclusions

We have demonstrated differences in the impact of cytoskeletal-regulating proteins on osteoblast adhesion formation, differentiation, and mineralization in response to substratum topography. While topographical manipulation osteoblasts can significantly affect differentiation, we demonstrate that pharmaceutical inhibition of ROCK activity acts synergistically with osteoconductive SLA to effectively speed osteoblast maturation, differentiation and mineralization. Further elucidation of the pathways through which adhesion and cytoskeletal changes regulate gene expression in osteoblasts will be instrumental in developing scaffolds and bioactive materials for bone tissue regeneration in future dental and orthopaedic applications.
